# A Structured Approach for Treating Calcium Hydroxylapatite Focal Accumulations

**DOI:** 10.1093/asj/sjae031

**Published:** 2024-02-16

**Authors:** Alec D McCarthy, Jani van Loghem, Keith A Martinez, Shino Bay Aguilera, David Funt

## Abstract

**Background:**

Radiesse, a widely utilized calcium hydroxylapatite (CaHA) dermal filler, has shown effectiveness in soft tissue augmentation and regeneration. As with all dermal fillers, the potential for nodules may arise. Understanding the pathogenesis of these nodules and exploring effective treatment methodologies are crucial for optimizing patient outcomes.

**Objectives:**

A literature search was carried out to identify published literature documenting reversal of CaHA nodules. After identification, a consensus panel developed a structured approach, denoted by levels, for applying such reversal methods.

**Methods:**

This concise review presents an algorithmic approach to addressing CaHA focal accumulations (noninflammatory nodules) based on invasiveness, cost, and potential risks based on published literature.

**Results:**

Level 0 involves no intervention, relying on natural degradation for asymptomatic nodules. Level 1 interventions utilize mechanical dispersion techniques, including massage and in situ dispersion, which have demonstrated high success rates, cost effectiveness, and minimal invasiveness. Level 2 introduces alternative modalities such as pharmacological treatments with 5-fluorouracil and corticosteroids, lasers, and experimental approaches. Level 3 represents last-resort options, including calcium-chelating agents, manual removal, and surgical excision.

**Conclusions:**

The article offers a structured approach to managing CaHA focal accumulations.

**Level of Evidence: 4:**

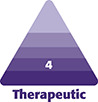

Radiesse (CaHA-CMC; Merz Aesthetics, Raleigh, NC), a calcium hydroxylapatite (CaHA) dermal filler and regenerative biostimulator, is widely employed in soft tissue augmentation by aesthetic providers for volumization, contouring, and soft tissue regeneration.^1,2^ With increasing utilization of dermal fillers, including CaHA and other particle-containing biostimulators like poly-l-lactic acid (PLLA) and polymethyl methacrylate, common adverse events have become more numerous, although not necessarily more frequent. Namely, subcutaneous nodules following injection are often cited as one of the most common unwanted complications to such treatments.^3^ These noninflammatory nodules (which are usually evident within several weeks following treatment), often characterized by focal accumulations of product, are commonly attributed to poor injection technique, suboptimal product spread, or excessive volume. A targeted approach is needed for their resolution.^4-6^

Understanding the underlying pathogenesis of particle-containing nodules and reviewing the published methodologies for their management is crucial for optimizing patient outcomes and effectively alleviating nodules. Poor injection technique, including improper needle placement, overfilling, or superficial injection, is postulated as the significant contributor of nodule formation. Focal accumulation nodules should be distinguished from delayed-onset nodules seen with hyaluronic acid fillers that appear to be immune responses that are product-, technique-, and patient-related in origin. Focal accumulation nodules should also be distinguished from granulomas, because they are not driven by an immunological response, but rather represent localized accumulations of CaHA microspheres. A comprehensive review of treatment options and their respective outcomes is imperative to guiding clinicians in selecting the most effective and safe approach for managing CaHA nodules.

Over the years, various methodologies have been proposed to disperse CaHA nodules, with different degrees of success. Conservative treatments such as massage and warm compresses have been attempted, but their efficacy remains limited, and they may not address the underlying microsphere accumulation and collagen scaffolding adequately. In contrast, an aqueous dispersal agent (ie, saline, lidocaine, hyaluronidase, sterile water, etc) has shown promise in some cases.^5^ In situ dispersion involves mechanical dispersal of the nodules at the injection site, aiming to distribute the CaHA microspheres more evenly within the surrounding tissues. This method has garnered attention for its potential to achieve favorable outcomes while being both cost effective and minimally invasive. To date, however, there exists a paucity of comprehensive literature evaluating the efficacy and safety of in situ dispersion compared to other treatment modalities. This review seeks to bridge this knowledge gap and give aesthetic providers evidence-based insights into the optimal management of CaHA nodules. After consolidating existing data and critically evaluating the published methodologies, an algorithmic management approach is presented ranging from the least (Level 1) to most (Level 3) invasive protocols.

##  

### Nodule Pathogenesis

Understanding the underlying mechanisms contributing to nodule formation in particle-based fillers is critical in their prevention and management. Nodules can be categorized as inflammatory (including granulomas) and noninflammatory nodules (accumulation of particles), the latter of which is seemingly most common.^4^ Nodules can also be categorized as delayed-onset nodules (DONs) (with onsets after several weeks or a few months) or focal accumulations (FAs), each of which have distinct characteristics.^3,4^ Notably, the presence or absence of lidocaine (Radiesse+ vs Radiesse), does not significantly influence the nodule formation rates in clinical trials.^7^

### Noninflammatory Nodules

Noninflammatory nodules may be characterized as focal accumulations of the filler material and are void of histiocytic and inflammatory cells histologically.^8^ They may clinically manifest as firm and nontender or tender, and may present with little or no erythema.^9^ Figure 1A depicts deeper, nontender focal accumulations on the jawline. Similarly, simple overfilling, while not nodules, may present as overcorrection that is visibly bothersome. Figure 1B depicts accidental superfluous deposition of CaHA-CMC near the cannula entry site and several superficial accumulations of CaHA-CMC. CaHA-CMC is unique in that it has both CaHA and carboxymethylcellulose (CMC) gel components. The CMC gel component usually resorbs in several months, whereas the CaHA components can persist for up to 2 years. It is conceivable that early-onset nodules may result from aggregations of CMC and/or CaHA microspheres. However, diluting CaHA-CMC past a 1:1 dilution effectively eliminates the occupying effect of the CMC gel, meaning that nodules arising in cases of dilute (1:1) or hyperdilute (1:>1) CaHA-CMC are almost always accumulations of the CaHA microspheres. This is visualized in the neck of a patient treated with hyperdilute CaHA-CMC (Figure 1C).^10^ Lin et al point out that the general causes of noninflammatory nodules are technique related and may result from injecting too quickly, injecting too large a volume of highly concentrated product, dislocation and accumulation of filler, injection of product too superficially, and injection into suboptimal locations (such as near sphincteric muscles).^6^ Figure 1D depicts an accumulation near the tear trough, while Figure 1E shows nodules from bolus injection into the mucosa, both potentially related to filler placement. When FAs are the cause of the nodule, early intervention is encouraged, because the CaHA component induces neocollagenesis and delaying treatment would allow collagen fiber deposition that could potentially further bind the CaHA microspheres together. In rare cases, delayed-onset, noninflammatory nodules may arise, particularly when CaHA is placed near or within sphincteric muscles, because their repeated contraction will gradually cause accumulation of the CaHA microspheres. Regardless, CaHA-CMC is composed of highly uniform CaHA microspheres, which may explain why their fraction of nodule complications is lower than that of PLLA, which is made of irregularly shaped, hydrophobic polymeric flakes that have larger surface areas for interacting and accumulating.^11-14^

**Figure 1.**
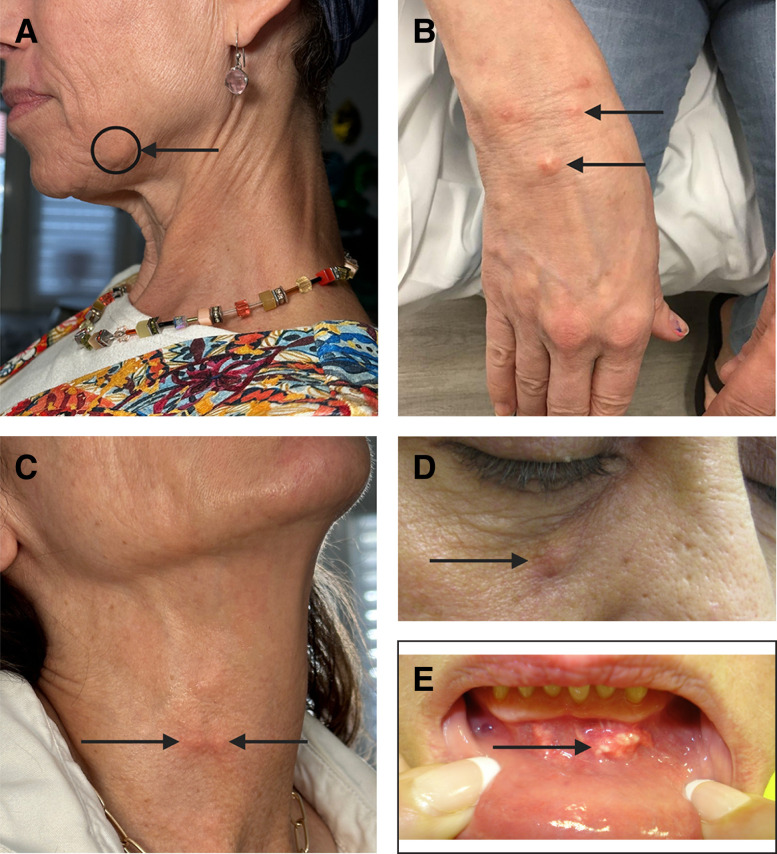
Examples of focal accumulations of CaHA in the (A, B) jawline and wrist of a 68-year-old female patient, (C) neck of a 63-year-old female patient, (D) tear trough of a 42-year-old female patient, and (E) oral mucosa of a 62-year-old female patient. CaHA, calcium hydroxylapatite.

### Inflammatory Nodules

In the case of inflammatory nodules, infection, biofilms, type IV hypersensitivity reactions, or granulomatous reactions may be the cause, particularly in nodules arising in proximity to infection or vaccination.^4,15,16^ These nodules often have delayed onsets and are characterized histologically by inflammatory cell infiltration, which is generally not observed as a normal clinical effect of CaHA-CMC.^17,18^ These nodules may present clinically as erythematous, tender, and edematous.^9^ Several factors may drive inflammatory or delayed-onset nodules, and understanding their pathogenesis should guide treatment paradigms. Infections or vaccinations are known contributors in DONs, a phenomenon that has been captured and articulated post-COVID.^15,19,20^ If infection is thought to be involved in nodule pathogenesis, it is generally recommended to deploy broad-spectrum antibiotics based on cultures.^9^ In cases in which nodules are recurrent without repeated injection, or persist following standard treatments described here, one may suspect biofilm formation, although it has been shown that the biofilm potential of CaHA-CMC is significantly lower than that of hyaluronic acid fillers.^4,21^ Regardless of their etiology, almost all approaches to delayed-onset or inflammatory nodules include antibiotic treatment, the inclusion of steroids, and/or 5-fluorouracil (5-FU) to inhibit fibroblast accumulation.^22^

## METHODS

Before determining the algorithm and determining the levels within the structure, a primary literature search was conducted by A.M., K.M., and J.V.L. Two databases, PubMed and Google Scholar, were searched from August to September 2023 for peer-reviewed publications on reversing nodules or granulomas of CaHA. Literature published after 2004 and in all languages was included in our search. Search terms are listed in Supplemental Table 1, available online at www.aestheticsurgeryjournal.com. After our search, 19 articles were identified and 8 were positioned in the structured algorithm. Articles containing discussion of sodium thiosulfate (STS), a calcium-chelating agent, are discussed but were excluded from inclusion in the treatment algorithm. A.M., K.M., J.V.L., S.B., and D.F. all deliberated on the order and inclusion of each article based on its scientific evidence, relevance to clinical practice, ease of use, rate of resolution, and potential for complication.

## RESULTS

### Applying the Algorithm

After distinguishing between noninflammatory and inflammatory nodules, the treating healthcare provider should assess whether an intervention is necessary and exercise deference to the patient. If the nodules are small and not visible, Level 0 is suggested. If the nodules require treatment, starting at Level 1 and progressing successively is suggested, based on the efficacy of the treatment. While these levels are for treating noninflammatory nodules, they may be applied to inflammatory nodules that have been delineated from the underlying pathological process. That is, once the inflammatory pathology is resolved, if any accumulations persist, these interventions may be applied.

#### Level 0—No Intervention, Natural Degradation

In many cases, nodules may be palpable but not visible. In such instances, nodules generally spontaneously resolve with time. The nodules generally do not outlive the lifespan of the material, which has been shown to completely resorb within 2.5 years.^23^ For example, Zhu and Cole report on a case of nodule formation in the cheek of a 72-year old patient that had persisted for 1 month. Noting that nodules from CaHA would resolve during degradation, the providers opted for no intervention and observed spontaneous resolution 4 months later.^24^ However, in such cases, it may be wise to defer to patient preference.

#### Level 1—Minimal Intervention

**Figure 2.**
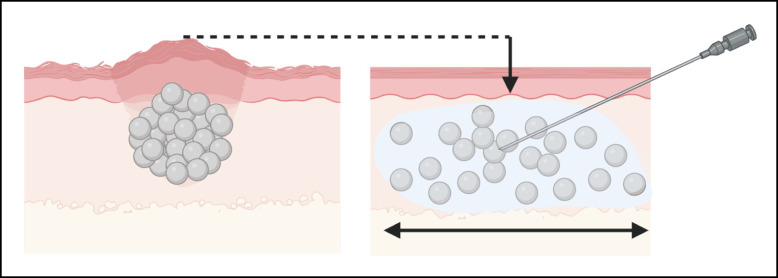
Illustration of a nodule composed of accumulated microspheres with vertical displacement of tissue being alleviated with in situ dispersion.

**Figure 3.**
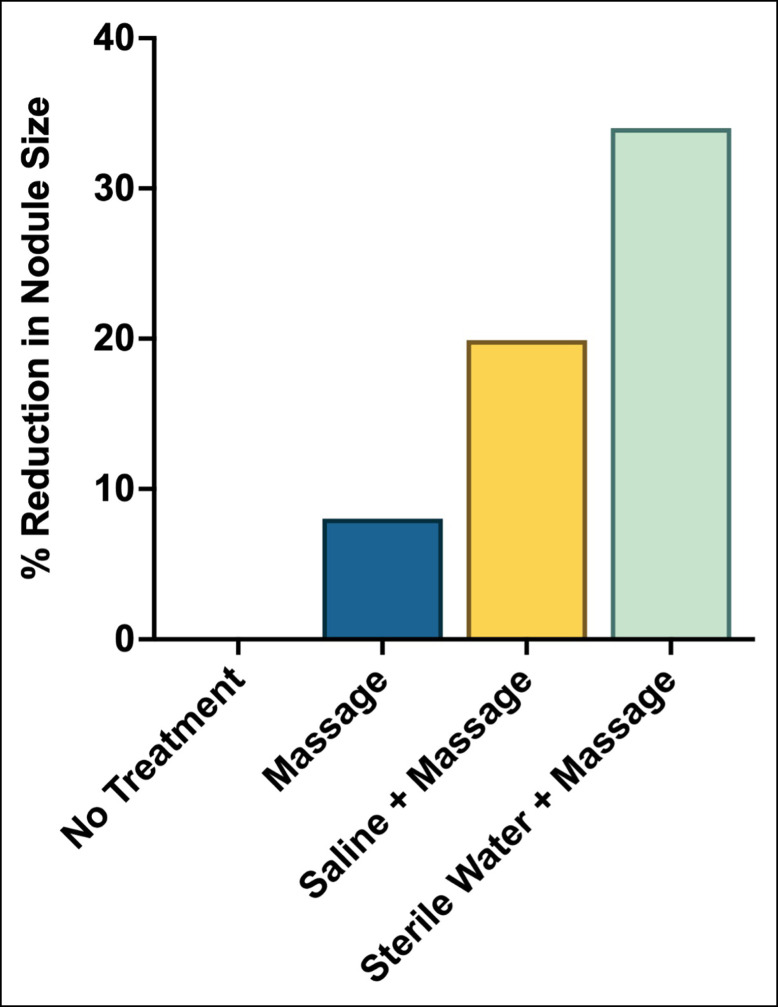
Reduction in nodule size following no treatment, massage only, dispersion with saline followed by massage, and dispersion with sterile water followed by massage. Reproduced with permission from Voigts et al 2010.

Level 1 interventions are the least invasive, costly, and risky. Arguably the most common method for reversing nodules is redistributing the accumulated material over a larger surface area. The concept behind dispersing nodules centers on creating an in situ hyperdilution and employing mechanical agitation to facilitate lateral and deep spread of the CaHA particles (Figure 2). It is well known that diluting CaHA effectively increases its spread, both to deep and lateral tissues.^25,26^ It is conceivable that dispersion will effectively spread particles to adjacent tissue, thereby alleviating the nodules. In the simplest terms, adding an aqueous solution at the site of the nodule provides a medium for particles to diffuse into. This diffusion can be further facilitated by mechanical agitation, such as vigorous massage. Voigts et al demonstrated the efficacy of simple dispersion with and without massage and administering saline and/or sterile water.^5^ Massage alone reduced the size of the nodules by approximately 10%, while saline and massage, and sterile water and massage, reduced the size of the nodules by approximately 20% and 35% respectively (Figure 3). The generalized protocol put forth by Voigts et al was as follows:

1. Create an in situ hyperdilution with sterile water or saline by injecting directly into the nodule.
2. Manual compression of the diluent bleb.
3. Vigorous massage immediately following injection of diluent.
4. Repeat if necessary.

When deploying the Voigts dispersal method, the addition of lidocaine will make the process more comfortable for the patient and increase compliance if multiple sessions of dispersal are required. Anecdotal reports suggest that replacing the saline or sterile water with hyaluronidase may enhance the dispersion of the nodule by dissolving endogenous hyaluronic acid that may interfere with in-plane hydrodissection. Therefore repeating the simple protocol suggested by Voigts et al but with hyaluronidase may enhance the treatment efficacy.

Another Level 1 approach, although not yet studied in a randomized fashion, expands on mechanical agitation to disperse the particles. Vibration from devices may act as a high-frequency adjuvant for massage. A simple approach utilizing topical microneedling has been observed to rapidly resolve nodules based on the following protocol (Video, available online at www.aestheticsurgeryjournal.com):

1. Preheat the tissue at the site of the nodule with warm compression.
2. Create an in situ hyperdilution with either sterile water, saline, or hyaluronidase.
3. Compress the diluent bleb.
4. With either radiofrequency or mechanized microneedling, perform several passes at depths ranging from 0.5 to 1.5 mm directly on and around the nodule.
5. Complete with manual massage.
6. Repeat if necessary.

**Figure 4.**
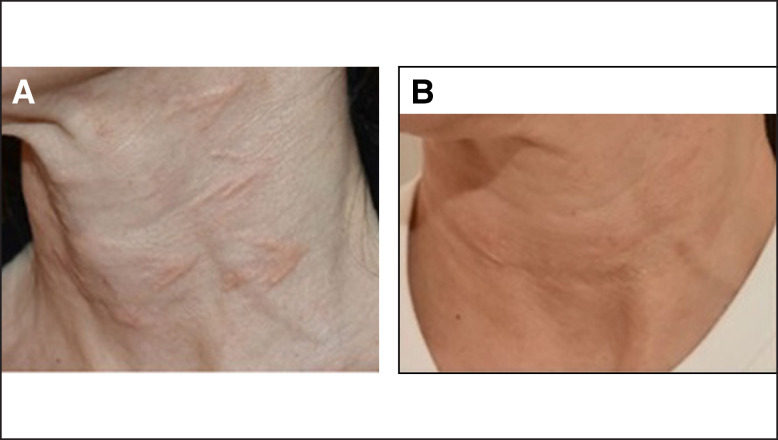
Images of noninflammatory nodules on a 45-year-old patient (A) one month after the initial treatment of hyperdilute CaHA-CMC in the neck and (B) 4 months after treatment with thermomechanical ablation. Reproduced with permission from Nipshagen et al 2020. CaHA, carboxylapatite; CMC, carboxymethylcellulose.

Nipshagen et al report a case of noninflammatory nodules developing from superficial placement of CaHA intradermally, which was confirmed with histology.^8^ Their primary interventions of topical triamcinolone cream 0.1% and oral prednisone followed by 3 sessions of intralesional STS failed. Following failure of primary and secondary interventions, the authors deployed 4 treatments of thermomechanical ablation with thermal-mechanical action (TMA Technology; Tixel, Sentient, Park City, UT) with some degree of success (Figure 4). Their successful deployment of a Level 1 mechanical-assisted dispersal was as follows:

1. The area with nodules was treated with TMA on mode 12/600.
2. The treatment was repeated 4 times.

Level 1 interventions are relatively low cost, minimally invasive, do not involve any pharmacologics, and have a high success rate, making them an ideal first-line treatment for nodules. Dispersion can be augmented with massage or vibration, and diluents can be sterile water, hyaluronidase, saline, lidocaine, or combinations of the 4.

#### Level 2—Pharmacological and Laser Interventions

Level 2 approaches utilize a variety of modalities that may be second-line approaches for nodules and may utilize a variety of devices or pharmacologics. Level 2 approaches may address focal accumulations or delayed-onset nodules. Aguilera et al presented a case report utilizing intralesional injections of 5-fluorouracil (5-FU), dexamethasone, and triamcinolone for treating noninflammatory nodules with the following rationale.^14^ The 5-FU is a pyrimidine analog that demonstrates an inhibitory effect on fibroblasts, as does triamcinolone.^27,28^ The inclusion of a corticosteroid was aimed at reducing the side effects of the 5-FU, which may include burning, pain, erythema, and hyperpigmentation. The dexamethasone was included to also exert a cytoprotective effect on fibroblasts. In addition to simply dispersing the nodule with the aqueous solution of 5-FU, triamcinolone, and dexamethasone, this approach also dampened cellular components of nodules. The protocol put forth by Aguilera et al was as follows:

1. Prepare 1.0 mL of 5-FU (50 mg/ml), 0.5 mL of dexamethasone (4 mg/mL), and 0.1 mL of triamcinolone (10 mg/mL).
2. A single 0.2-mL injection of the solution was placed directly into the 1.3-cm nodule.
3. The ratio for injection was 0.15 mL solution to 1 mL nodule volume.

**Figure 5.**
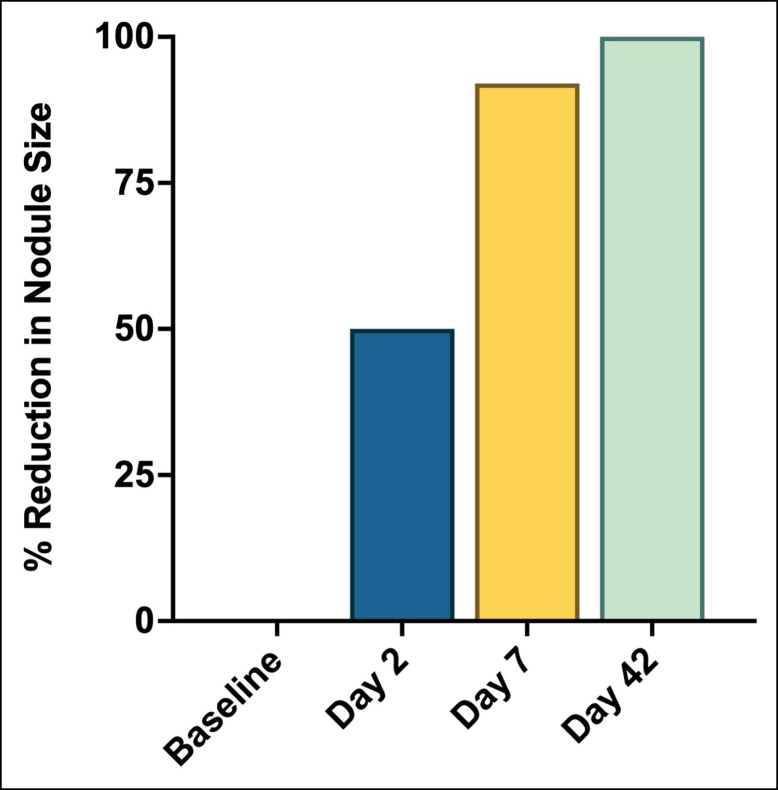
Reduction in nodule size following treatment with 5-FU, dexamethasone, and triamcinolone. 5-FU, 5-fluorouracil.

The authors observed an immediate reduction of 50% within 24 hours and total resolution after 6 weeks (Figure 5). It is again feasible that additional massage following injection of the solution may enhance the results of the treatment.

Another Level 2 approach utilizes lasers to resolve nodules. Lasers remain minimally invasive, and their application in reducing filler-induced nodules is well documented.^29,30^ In the case of Reddy et al, it was hypothesized that the laser assisted in particle dispersion. Non-aesthetic instances were cited from the literature.^30,31^ Reddy et al proposed the following protocol:

1. A fractional carbon dioxide laser with a 135-µm, 30 mJ energy and treatment level 8 (30% coverage) was utilized.
2. 7% lidocaine–7% tetracaine cream was applied before the procedure.
3. Prednisone (60 mg) was administered orally, and ketorolac tromethamine (30 mg; Pfizer, New York, NY) was administered by intramuscular injection.
4. The area of the nodule was treated with the laser.

After 2 weeks, the patient observed total and permanent resolution of the nodules, with the additional observation that skin laxity had been improved.

A similar protocol with an Erbium YAG laser, as reported by Vrcek et al, reported 2 cases of superficially placed CaHA resolved with a combination of dispersal and Erbium YAG lasering.^32^ Erbium YAG lasers, compared to CO2 lasers, are generally less aggressive and have fewer side effects, potentially making them a more suitable option. The protocol presented by Vrcek et al consisted of the following:

1. The nodules were injected with 0.5 cc of warmed bacteriostatic normal saline in a fanning pattern.
2. The area was treated with a nonablative, 1540-nm wavelength, fractional Erbium YAG laser to heat the saline.
3. The settings were: 15-millisecond pulse duration, 8 mJ energy, spot size of 15 mm, and 5 pulses per treatment.
4. Steps 1 to 4 were repeated for 5 sessions.
5. Any peripheral telangiectasias were treated with a potassium-titanyl-phosphate laser.

After 3 months, both patients had total resolution, with no side effects.

Approaches with lasers, although more costly, may have some advantages; the lasering procedure has inherent benefits to the skin and overall aesthetics of the patient, and the complications are minimal. However, the recovery and downtime from lasering procedures may be a drawback. Further, the efficacy of the treatment may vary based on the thickness of the skin at the location of the undesired filler material and may be limited by the penetrative depth of the laser.

Another Level 2 protocol is an experimental study conducted by Aksenenko et al and investigates devices (ultraphonophoresis, with an ultrasound device that maximizes the effects of a topically applied drug) and pharmacologics (collagenase and STS).^33^ Uniquely, this protocol is suggested for delayed treatment of nodules with the observation that CaHA effectively produces collagen. It is proposed that delayed treatment of nodules may allow ample time for de novo collagen to accumulate and “anchor” neighboring microspheres to one another, making dispersion particularly difficult. In this study, patients were divided into 3 groups: Group 1 was aligned with a Level 2 approach and consisted of treatment with ultraphonophoresis of collagenase (Collalysin 1000 CU) in a course of 10 procedures. Groups 2 and 3 aligned with Level 3 and will be discussed in the following section. In line with Level 2 (Group 1 in the study), Aksenenko et al proposed the following protocol with just ultraphonophoresis and collagenase:

1. Collagenase (Collalysin 1000 CU) was diluted in 2 to 3 mL of gel for ultrasound therapy (Repack-T gel).
2. The gel solution was placed on the nodule area.
3. The affected area was treated at an intensity of 0.2 to 0.4 W/cm^2^ under a continuous mode of operation.
4. The duration of the treatment was 5 to 8 minutes daily.
5. The procedure was repeated for the following 9 days (10 total treatments).

The mean volume of the nodules in Group 1 decreased from 0.816 cm^3^ to 0.778 cm^3^ by 3 months after the treatment series. There are several distinct advantages and disadvantages with this approach. First, it is noninvasive and requires minimal downtime or recovery. Second, it offers a unique approach to particularly stubborn nodules with an abundance of surrounding collagen. The drawbacks to this approach include the indiscriminate action of the collagenase on surrounding collagen as well as the necessity for daily treatment.

#### Level 3—Physical Removal

Level 3 approaches include manual removal or surgical excision, and in general are viewed as a last resort for treating nodules. It is important to note that nodules from CaHA will spontaneously resolve over time, and the authors encourage providers to reserve Level 3 interventions for last-resource scenarios. Manual removal methods should be selected based on the quantity and size of the nodules. Kim et al proposed a simple method for removing CaHA nodules with a needle or blade.^34^ The protocol suggested was as follows:

1. The nodule site was cleaned before treatment.
2. At the site of the nodule, a puncture was made with a needle or blade.
3. The filler was drained from the port (puncture site).
4. The port was covered with a bandage and kept clean for several days following extraction.

A similar approach was presented by Cohen et al and utilized a negative pressure microliposuction cannula for extraction.^35^ Cohen et al proposed the following straightforward protocol for debulking or removal of excess filler or nodules:

1. The area of treatment was sterilized with alcohol or a Betadine (Purdue Pharma, Stamford, CT) swab.
2. A small skin incision was made with an 18-gauge needle.
3. A 1-mm diameter, grater-type microliposuction cannula (Lipocube Inc., London, UK) was attached to a 5- to 10-mL syringe and introduced through the incision.
4. Negative pressure was created by withdrawing the syringe plunger.
5. The cannula was then moved from the base of the nodule superficially in a back-and-forth motion until the filler was removed.
6. Patients were given arnica for bruising and an ice pack was applied.

Cohen et al noted that all patients had resolution of excess filler or nodules after a single treatment. Although invasive, this approach is practical for removal of large volumes of CaHA and seemingly does not require repeat treatments for resolution. Unfortunately, permanent removal of the patient's fat surrounding the nodule is possible with this method.

## DISCUSSION

### Sodium Thiosulfate

Sodium thiosulfate has been well-studied as a reversal agent for CaHA based on its calcium-chelating action. STS is an inorganic sodium salt that treats heavy metal exposure in humans. STS is thought to potentially extract the calcium component from the CaHA, thereby gradually dissolving the microspheres. The first instance of dissolution of CaHA particles with STS was reported by Kreymereman et al in mini-pigs.^36^ Following this initial report, a series of studies were conducted and cases reported, including that from Aksenenko et al, with varying degrees of reported success.^33,37,38^

**Figure 6.**
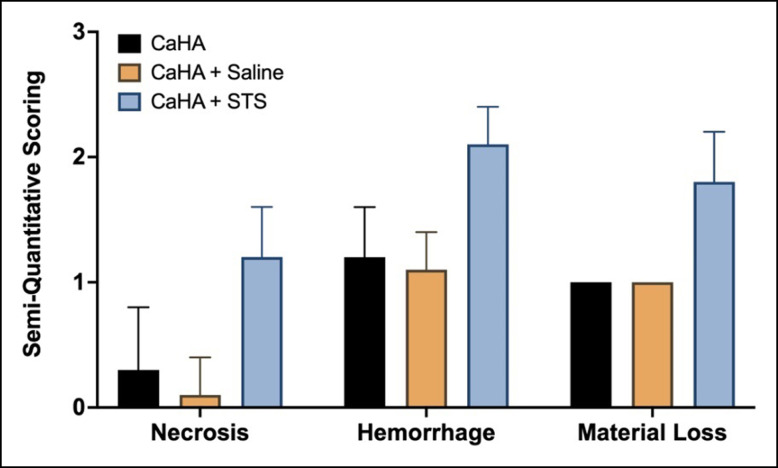
Necrosis, hemorrhage, and material loss scoring on nodules treated with nothing, saline, and sodium thiosulfate as reported by Danysz et al. CaHA, calcium hydroxylapatite; STS, sodium thiosulfate.

Despite data demonstrating some efficacy with STS, a controlled study has shed light on the potential mechanism of action of STS. A thorough preclinical study by Danysz et al investigated STS in a pig model with 3-dimensional camera analysis, micro–computed tomography, computed tomography in vivo, scanning electron microscopy, and histopathology to evaluate the true effect of STS.^39^ In this study, there were no observations of CaHA microsphere degradation from STS. In this case, STS did not outperform water or phosphate-buffered solution under any conditions or at any time points. Additionally, the volume of the nodules varied only marginally. The authors concluded that the primary mechanism of STS was a dispersing effect, consistent with that reported by Voigts et al, and further noted that STS carried the risk of tissue necrosis and hemorrhage, whereas saline or sterile water did not (Figure 6). Evidence of STS functioning as a nodule reversal agent is best captured in Nipshagen et al's report of superficial CaHA failing to respond to STS, postulating that this was due to the lack of tissue volume for dispersal.^8^ It is worth noting that another study found that STS-treated nodules dispersed the same distance as saline-treated nodules.^40^

Noting the potential risks of necrosis and hemorrhage, the lack of efficacy in controlled studies, and the mechanism of action, the authors do not recommend STS as a reversal agent for CaHA nodules.

### Commentary on Treatments

Several comments on the treatment of nodules are warranted here. The authors suggest that mechanical dispersion of the particles is the most effective and least-burdensome approach for reversing nodules. Similarly, in a symptomatic visible nodule, treatment should not be delayed, because early treatment, before significant neocollagenesis, is generally easier. It is conceivable that devices that impart vibration (ie, vibratory tools, microneedles, etc) may have a place in treating nodules. Second, the role of mechanical subcision is not to be understated. Third, precisely locating nodules with ultrasound may warrant consideration, particularly with intralesional 5-FU, STS, or collagenase, because these compounds should be utilized sparingly. It is also conceivable that these protocols can be combined or applied in conjunction, similar to the dispersion + lasering method reported by Vrcek et al.^32^ For example, adding vibration or mechanical disruption to a sterile water– or hyaluronidase-diluted nodule after subcision would theoretically capitalize on the benefits of all treatments.

### Summary

**Figure 7.**
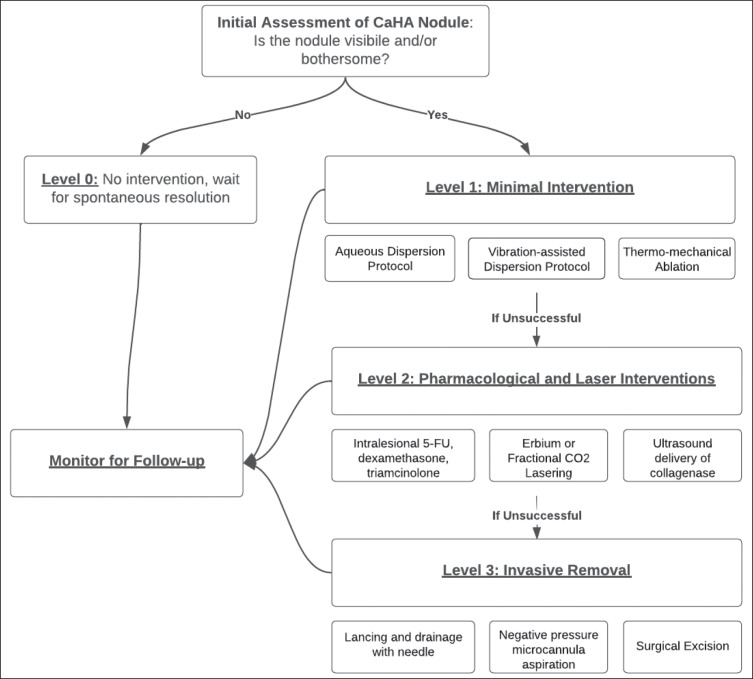
Flow chart for managing focal accumulations of CaHA. CaHA, calcium hydroxylapatite.

The management of CaHA nodules presents a challenging and crucial aspect of aesthetic medicine. The leveled approach categorizes treatment modalities based on their invasiveness, cost, and potential risks, providing clinicians with evidence-based insights into the optimal management of CaHA nodules. The overall flow for treatment progression is visualized in Figure 7.

Level 0, which involves no intervention and relies on natural degradation, may be suitable for cases in which nodules are palpable but not visible. In such instances, nodules are likely to resolve over time as the CaHA naturally degrades. Patient preference plays a significant role in deciding whether to intervene in such cases.

Level 1 interventions represent the least invasive and least costly approaches, with a high success rate. Dispersion techniques, including massage and in situ dispersion, have shown promise in reducing nodule size and can be augmented by continued at-home massage from the patient. Additionally, mechanical agitation through topical microneedling or vibration devices has been observed to resolve nodules rapidly. Administration of hyaluronidase in conjunction with dispersion techniques may further enhance treatment efficacy.

Level 2 interventions involve a variety of modalities that may be considered second-line approaches for treating nodules, especially in the case of delayed-onset nodules or long-standing nodules. Intralesional injections of 5-fluorouracil (5-FU), dexamethasone, and triamcinolone have been suggested as a pharmacological treatment option. Lasers, particularly fractional carbon dioxide lasers, have also been effective in reducing filler-induced nodules, with additional skin rejuvenation benefits. An experimental study with ultraphonophoresis and collagenase or STS has shown potential for particularly stubborn nodules anchored by surrounding collagen.

Level 3 interventions are considered the last resort for treating nodules and include calcium-chelating agents, manual removal, and surgical excision. STS has been extensively studied as a potential treatment for CaHA nodules, but its mechanism of action remains unclear. Although some studies have reported success with STS, others have shown limited efficacy in dissolving CaHA microspheres. Manual removal methods may be suitable for larger volumes of CaHA, but they are more invasive and carry inherent risks from surgery.

Overall, mechanical dispersion techniques have demonstrated consistent efficacy and should be considered first-line treatments for CaHA nodules. Levels 2 and 3 interventions may be considered for specific cases in which other treatments have failed or for particularly challenging nodules. The selection of treatment modality should be individualized based on the characteristics of the nodules, patient preferences, and the expertise of the clinician. It is important to note that this review provides a comprehensive assessment of the available treatment options, but the evidence may evolve as new studies and clinical experiences emerge. Continued research is essential to further refining and optimizing the management of CaHA nodules and enhancing patient outcomes in the field of aesthetic medicine.

## CONCLUSIONS

In conclusion, managing CaHA nodules in aesthetic medicine requires a comprehensive understanding of their underlying pathogenesis and a critical review of treatment options. The leveled approach presented in this concise review provides evidence-based insights for clinicians. Level 0 involves natural degradation and may be suitable for asymptomatic nodules. Level 1 interventions, such as mechanical dispersion techniques, are preferred due to their efficacy, safety, and cost effectiveness. Level 2 interventions offer alternative modalities, including pharmacological treatments and lasers, while Level 3 represents last-resort options like manual removal and surgical excision. Although this article is intended to guide clinical decision-making on CaHA focal accumulation reversal, it is worth pointing out that there is no reversal agent for CaHA, and therefore continued research in this area is warranted. The field of aesthetic medicine is continually evolving, necessitating ongoing research to optimize CaHA nodule management and enhance patient outcomes.

## Supplemental Material

This article contains supplemental material located online at www.aestheticsurgeryjournal.com.

## Supplementary Material

sjae031_Supplementary_Data
