# Supplementary material for: A Structured Approach for Treating Calcium Hydroxylapatite Focal Accumulations
Source: Aesthet Surg J. 2024 Feb 16;44(8):869–79. doi: 10.1093/asj/sjae031 (PMC11333958; doi:10.1093/asj/sjae031)
Supplement: sjae031_Supplementary_Data [file sjae031_Supplementary_Data.zip › Supplemental Table 1.docx]

**Supplemental Table 1.** Search Terms Included in Our Review

| Term + nodule | Term + nodule reversal | Term + nodule treatment |
| --- | --- | --- |
| Radiesse | Radiesse | Radiesse |
| CaHA | CaHA | CaHA |
| calcium hydroxylapatite | calcium hydroxylapatite | calcium hydroxylapatite |
| Calcium hydroxyapatite | Calcium hydroxyapatite | Calcium hydroxyapatite |
| Radiance | Radiance | Radiance |
| CaHA-CMC | CaHA-CMC | CaHA-CMC |
| Harmonyca | Harmonyca | Harmonyca |
